# Sequential Serous Choroidal Detachment in Subjects Undergoing Bilateral Trabeculectomy

**DOI:** 10.18502/jovr.v19i4.13881

**Published:** 2024-12-31

**Authors:** Maryam Yadgari, Kiana Hassanpour, Fatemeh Vafaei, Nina Firoozian, Maryam Oraee Yazdani, Zahra Khorrami, Sadid Hooshmandi

**Affiliations:** ^1^Ophthalmic Research Center, Research Institute for Ophthalmology and Vision Science, Shahid Beheshti University of Medical Sciences, Tehran, Iran; ^2^Basir Eye Health Research Center, Iran University of Medical Sciences, Tehran, Iran; ^3^Ophthalmic Epidemiology Research Center, Research Institute for Ophthalmology and Vision Science, Shahid Beheshti University of Medical Sciences, Tehran, Iran; ^5^https://orcid.org/0000-0003-0829-1861; ^6^https://orcid.org/0000-0001-5079-7239

**Keywords:** Bilateral, Serous Choroidal Detachment, Trabeculectomy

## Abstract

**Purpose:**

This study aims to assess the incidence of serous choroidal detachment (SCD) in the second eye of patients undergoing bilateral trabeculectomy (BT) and evaluate its impact on the clinical outcomes and failure rate of trabeculectomy in the second-operated eyes.

**Methods:**

This retrospective case–control study analyzed 90 eyes of 45 patients who underwent BT. Surgical success was defined as maintaining intraocular pressure (IOP) between 5 and 21 mmHg, requiring no additional glaucoma surgery, and exhibiting a visual acuity of at least light perception. Relevant patient data, such as age, glaucoma type, systemic diseases, preoperative and postoperative IOP, and complications, were extracted from medical records.

**Results:**

The mean age of patients was 59.8 ± 11.1 years. The five-year cumulative probability of success in the first- and second-operated eyes was 61.0% and 67.6%, respectively (log rank = 0.085, *P* = 0.77). Among the participants, 28.9% experienced SCD, and 76.9% of those who had SCD in the first-operated eye developed the same condition in the second eye as well (*P* < 0.001). In the first-operated eyes, the five-year cumulative probability of survival was 71.7% without SCD and 35.0% with SCD (log rank = 2.59, *P* = 0.107).

**Conclusion:**

The occurrence of SCD in the first eye following trabeculectomy may indicate a predisposition to its development in the second eye during BT. Furthermore, the surgical success rate of the second-operated eye is comparable to the outcomes of the first eye undergoing BT.

##  INTRODUCTION

Glaucoma stands as a leading contributor to irreversible blindness, characterized by the gradual loss of retinal ganglion cells.^[1]^ Globally, it affects more than 70 million individuals, and about 10% of this population confront significant bilateral vision impairment.^[2]^

The primary objective in the management of glaucoma is preventing disease progression and preserving the quality of life for affected individuals.^[3]^ The main therapeutic intervention is reducing the intraocular pressure (IOP).^[4]^ This treatment approach typically commences with medical therapy and progresses to surgical alternatives for patients exhibiting refractory glaucoma.^[5]^

Trabeculectomy, introduced over five decades ago, stands as one of the most frequently undertaken procedures for the surgical management of refractory glaucoma, particularly common in developing countries.^[6]^ The reported success rate of trabeculectomy spans from 48% to 98%, depending on the definition of success and the duration of follow-up.^[7]^ Nevertheless, it is imperative to acknowledge that trabeculectomy is linked to a higher risk of complications in comparison to alternative procedures.^[8]^

Various studies have identified several risk factors influencing the success rate of trabeculectomy, encompassing patient's age, glaucoma type, preoperative medication, and the severity of glaucoma.^[9]^

Moreover, the occurrence of intra- and postoperative complications can exert a discernible impact on the overall success rate of the surgical intervention. Since glaucoma often affects each eye differently, surgical interventions may be necessary for both eyes, but typically at different times.^[10,11]^ The conventional approach involves prioritizing the eye with more pronounced vision loss and advanced glaucoma for the initial procedure.^[12]^ Notably, research indicates that the success rate of trabeculectomy in the first-operated eye can serve as a predictive indicator for the success rate in the contralateral eye.^[12,13]^

Recognizing the risk factors associated with failure in the second eye can provide valuable insights for glaucoma surgeons, enabling them to make more informed decisions about surgical interventions for patients.

Our study sought to examine the occurrence of serous choroidal detachment (SCD) in the contralateral eye following bilateral trabeculectomy (BT). Furthermore, we evaluated the clinical outcomes and factors influencing the success of trabeculectomy in the second-operated eye among patients undergoing BT.

##  METHODS

In this retrospective case–control study, all patients deemed refractory to maximum tolerated medication, that is, the highest level of drug therapy a patient can endure without intolerable side effects or significant adverse reactions even if it fails to achieve the desired treatment outcome, underwent BT at an academic tertiary ophthalmology center between January 2015 and July 2020. The exclusion criteria comprised glaucoma secondary to uveitis, trauma, or neovascular etiologies, and individuals with a history of previous intraocular surgery (excluding uncomplicated cataract surgery). The study adhered to the principles of the Declaration of Helsinki and was approved by the Ethics Committee of Shahid Beheshti University of Medical Sciences (IR.SBMU.MSP.REC.1400.785).

### Surgical Technique

Both eyes of each patient underwent surgical procedures by the same glaucoma specialist (MY). A fornix-based conjunctival incision was performed in all procedures, accompanied by the creation of a triangular or rectangular scleral flap situated approximately 4 × 3 mm posterior to the limbus and 1 mm to the clear cornea. A sponge soaked in 0.02% mitomycin C was applied over the scleral flap for a duration of 2 minutes, followed by meticulous irrigation with a balanced salt solution. Subsequent to the creation of a paracentesis, sclerotomy and iridectomy were executed. The scleral flap and conjunctiva were closed using 10-0 nylon sutures in an interrupted fashion. The postoperative regimen included chloramphenicol eye drops (Chlobiotic, Sina Darou, Tehran, Iran) administered four times daily for one week, alongside betamethasone eye drops (Betasonate, Sina Darou, Tehran, Iran), also given four times daily and tapered based on conjunctival hyperemia. Glaucoma medication would be initiated if IOP surpassed the range of 15 to 21 mmHg, in accordance with the target pressure. Postoperative management was consistently overseen by an experienced glaucoma surgeon (MY), who performed slit-lamp biomicroscopy, Goldmann applanation tonometry, and dilated fundoscopy conducted at each follow-up visit.

### Data Gathering

Patient records were thoroughly evaluated with respect to pre- and postoperative IOP, glaucoma type, administered glaucoma medication(s), and systemic conditions such as diabetes mellitus, hypertension, and ischemic heart disease. The data pertaining to intra- and postoperative complications were also extracted and analyzed.

### Statistical Analysis

Data were presented as mean, median, and standard deviation. The normality of data was assessed using Shapiro-Wilk test. Parametric and non-parametric tests, including *T*-test, chi-square, Fisher exact test, Mann–Whitney test, and Wilcoxon signed-rank test, were employed for univariate analysis. A generalized estimating equation (GEE) was utilized to conduct multiple comparisons, adjusting for the correlation between the two eyes. Kaplan-Meier analysis was applied to depict survival rates in the total, first- and second-operated eyes. Success was defined as achieving an IOP < 21 mmHg (i.e., a reduction of at least 20% from the baseline IOP), no need for further glaucoma surgery during the follow-up period, and no total loss of vision. All statistical analyses were performed using SPSS version 25.0 (IBM, SPSS Inc, Chicago, Illinois).

In the post hoc analysis, employing the observed hazard ratio of 5.3 for SCD, we obtained a study power of 91% at a significance level of 0.05. This calculation was performed using G*Power software in order to ensure that our sample size possessed adequate power to detect significant effects.

**Figure 1:**
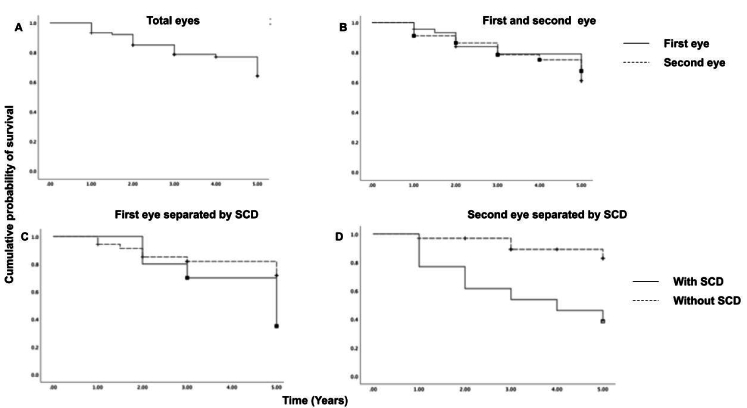
Kaplan-Meier survival plot demonstrates the cumulative probability of success rates between the study groups: (A) in all eyes; (B) between the first- and second-operated eyes; (C) in the first-operated eyes in patients with serous choroidal detachment (SCD) and without SCD; (D) in the second-operated eyes in patients with SCD and without SCD.

**Figure 2:**
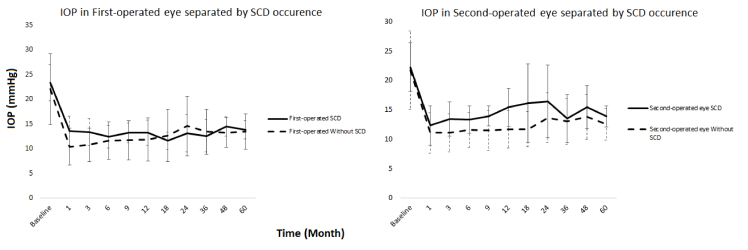
IOP diagrams over the study period. (A) IOP in first-operated eyes in patients with SCD and patients without SCD. (B) IOP in second-operated eyes in patients with SCD and patients without SCD. IOP, intraocular pressure; SCD, serous choroidal detachment.

**Table 1:** Patient's baseline characteristics.

	**Patients with SCD**	**Patients without SCD**	** P-value**
Age (mean ± SD)	59.61 ± 12.38	59.94 ± 10.9	0.932
Male	11 (84.6%)	19 (59.4%)	0.165
Female	2 (15.4%)	13 (40.1%)	
Visual acuity (LogMAR)	1.03 ± 0.12	0.88 ± 0.43	0.649
First-operated eye			
Right	4 (30.8%)	18 (56.2%)	0.203
Left	9 (69.2%)	14 (43.8%)	
Type of glaucoma (patients)			0.261
POAG	10 (43.5%)	31 (46.3%)	
CACG	4 (17.4%)	19 (28.4%)	
PEG	9 (39.1%)	9 (13.4%)	
others (FHI, PDS...)	0 (0%)	8 (11.9%)	
Diabetes mellitus	1	5	0.171
SCD, serous choroidal detachment; POAG, primary open-angle glaucoma; CACG, chronic angle closure glaucoma; PEG, pseudoexfoliation glaucoma; FHI, Fuchs heterochromic iridocyclitis; PDS, pigment dispersion syndrome; SD, standard deviation			

**Table 2:** The hazard ratio of the factors affecting the survival of the second-operated eye.

**Variable **	**HR**	** P-value**
Age	0.98	0.63
SCD	5.3	0.006
Baseline IOP	1.1	0.05
Baseline medication	2.7	0.05
Sex	1.5	0.63
Type of glaucoma	1.6	0.38
The time between the surgeries	1.4	0.25
Result of the first surgery	0.93	0.9
SCD, serous choroidal detachment; IOP, intraocular pressure; HR, hazard ratio		

**Table 3:** IOP and glaucoma medication in all patients and separated by first- and second-operated eyes.

	Total ${\;}^{**}$	**First-operated eye ${\;}^{\$}$ **	**Second-operated eye ${\;}^{\$}$ **	P-value ${\;}^{*}$
Preoperative				
IOP	22.07 ± 6.28	22.31 ± 6.60	21.84 ± 6.01	0.73
Number of medications	3.20 ± 0.69	3.16 ± 0.67	3.25 ± 0.72	0.52
1 M				
IOP	11.25 ± 3.57	11.11 ± 3.79	11.40 ± 3.39	0.70
Number of medications	0.11 ± 0.48	0.11 ± 0.49	0.11 ± 0.49	1.0
*P*-value within groups	< 0.001	< 0.001	< 0.001	
3 M				
IOP	11.53 ± 3.32	11.33 ± 3.38	11.73 ± 3.28	0.57
Number of medications	0.34 ± 0.72	0.33 ± 0.74	0.36 ± 0.71	0.88
*P*-value within groups	< 0.001	< 0.001	< 0.001	
6 M				
IOP	11.93 ± 3.18	11.8 ± 3.5	12.1 ± 2.9	0.59
Number of medications	0.48 ± 0.88	0.42 ± 0.81	0.53 ± 0.94	0.55
*P*-value within groups	< 0.001	< 0.001	< 0.001	
9 M				
IOP	12.11 ± 3.41	12.04 ± 3.67	12.18 ± 3.18	0.85
Number of medications	0.71 ± 1.08	0.69 ± 1.02	0.73 ± 1.16	0.85
*P*-value within groups	< 0.001	< 0.001	< 0.001	
12 M				
IOP	12.43 ± 3.82	12.11 ± 4.05	12.76 ± 3.60	0.43
Number of medications	1.14 ± 1.28	1.20 ± 1.31	1.09 ± 1.26	0.68
*P*-value within groups	< 0.001	< 0.001	< 0.001	
18 M				
IOP	12.62 ± 4.71	12.45 ± 4.89	12.79 ± 4.58	0.75
Number of medications	1.17 ± 1.25	1.15 ± 1.23	1.21 ± 1.28	0.85
*P*-value within groups	< 0.001	< 0.001	< 0.001	
24 M				
IOP	14.32 ± 5.25	14.33 ± 5.69	14.33 ± 4.85	0.99
Number of medications	1.44 ± 1.29	1.45 ± 1.24	1.44 ± 1.35	0.96
*P*-value within groups	< 0.001	< 0.001	< 0.001	
36 M				
IOP	13.18 ± 4.11	13.24 ± 4.35	13.12 ± 3.89	0.90
Number of medications	1.98 ± 1.22	2.07 ± 1.23	1.89 ± 1.22	0.59
*P*-value within groups	< 0.001	< 0.001	< 0.001	
48 M				
IOP	13.91 ± 3.33	13.57 ± 2.83	14.25 ± 3.79	0.48
Number of medications	2.23 ± 1.04	2.26 ± 1.05	2.21 ± 1.06	0.86
*P*-value within groups	< 0.001	< 0.001	< 0.001	
60 M				
IOP	13.27 ± 2.85	13.55 ± 3.17	12.95 ± 2.48	0.51
Number of medications	2.17 ± 1.16	2.14 ± 1.21	2.21 ± 1.13	0.84
*P*-value within groups	< 0.001	< 0.001	< 0.001	
Data are shown in mean ± standard deviation; IOP, intraocular pressure; M, month ${\;}^{*}$ Based on paired *T*-test between first- and second-operated eyes; ${\;}^{**}$ Based on the generalized estimating equation; ${\;}^{\$}$ Based on the generalized linear model.				

**Table 4:** IOP and glaucoma medication in the first- and second-operated eyes in patients with SCD and without SCD.

	**First-operated eye** ${\;}^{**}$			**Second-operated eye** ${\;}^{**}$		
	**Patients without SCD**	**Patients with SCD**	***P*** -value ${\;}^{*}$	**Patients without SCD**	**Patients with SCD**	***P*** -value ${\;}^{*}$
Preoperative						
IOP	22.03 ± 7.24	23.30 ± 3.68	0.46	21.69 ± 6.68	22.23 ± 4.11	0.79
Number of medications	3.14 ± 0.65	3.20 ± 0.79	0.82	3.19 ± 0.70	3.38 ± 0.77	0.43
1 M						
IOP	10.40 ± 3.74	13.60 ± 2.91	0.02	11.03 ± 3.39	12.31 ± 3.33	0.26
Number of medications	0.14 ± 0.55	0.00 ± 0.00	0.42	0.16 ± 0.57	0.00 ± 0.00	0.33
*P*-value within groups	< 0.001	0.009		< 0.001	0.002	
3 M						
IOP	10.77 ± 3.36	13.30 ± 2.75	0.03	11.06 ± 3.23	13.38 ± 2.90	0.03
Number of medications	0.29 ± 0.75	0.50 ± 0.71	0.42	0.22 ± 0.61	0.69 ± 0.85	0.04
* P*-value within groups	< 0.001	0.011		< 0.001	0.003	
6 M						
IOP	11.6 ± 3.8	12.4 ± 2.3	0.51	11.6 ± 3.0	13.3 ± 2.3	0.07
Number of medications	0.37 ± 0.84	0.60 ± 0.70	0.44	0.31 ± 0.74	1.08 ± 1.19	< 0.001
* P*-value within groups	< 0.001	0.007		< 0.001	0.002	
9 M						
IOP	11.71 ± 3.98	13.20 ± 1.99	0.26	11.47 ± 3.39	13.92 ± 1.66	0.02
Number of medications	0.63 ± 1.03	0.90 ± 0.99	0.46	0.50 ± 1.05	1.31 ± 1.25	0.03
* P*-value within groups	< 0.001	0.007		< 0.001	0.002	
12 M						
IOP	11.80 ± 4.37	13.20 ± 2.53	0.34	11.69 ± 3.19	15.38 ± 3.28	0.001
Number of medications	1.09 ± 1.31	1.60 ± 1.26	0.28	0.62 ± 0.98	2.23 ± 1.17	< 0.001
* P*-value within groups	< 0.001	0.007		< 0.001	0.003	
18 M						
IOP	12.64 ± 5.33	11.57 ± 1.81	0.61	11.66 ± 3.02	16.10 ± 6.62	0.07
Number of medications	1.09 ± 1.18	1.43 ± 1.51	0.52	0.79 ± 1.01	2.40 ± 1.26	< 0.001
* P*-value within groups	< 0.001	0.017		< 0.001	0.092	
24 M						
IOP	14.58 ± 6.03	13.14 ± 3.80	0.55	13.62 ± 4.19	16.40 ± 6.19	0.12
Number of medications	1.30 ± 1.21	2.14 ± 1.21	0.10	1.00 ± 1.16	2.70 ± 1.06	< 0.001
* P*-value within groups	< 0.001	0.017		< 0.001	0.056	
36 M						
IOP	13.40 ± 4.59	12.57 ± 3.31	0.66	13.00 ± 3.92	13.50 ± 4.04	0.76
Number of medications	2.00 ± 1.24	2.29 ± 1.25	0.60	1.60 ± 1.23	2.71 ± 0.76	< 0.001
* P*-value within groups	< 0.001	0.016		< 0.001	0.017	
48 M						
IOP	13.24 ± 3.05	14.50 ± 1.97	0.36	13.76 ± 3.83	15.43 ± 3.69	0.34
Number of medications	2.18 ± 1.19	2.50 ± 0.55	0.53	1.94 ± 1.14	2.86 ± 0.38	0.008
* P*-value within groups	< 0.001	0.026		< 0.001	0.143	
60 M						
IOP	13.44 ± 3.60	13.83 ± 1.83	0.80	12.54 ± 2.70	13.83 ± 1.83	0.24
Number of medications	2.00 ± 1.37	2.50 ± 0.55	0.23	1.92 ± 1.26	2.83 ± 0.41	0.03
* P*-value within groups	< 0.001	0.046		0.001	0.056	
Data are shown in mean ± standard deviation; SCD, serous choroidal detachment; IOP, intraocular pressure; M, month ${\;}^{*}$ Based on the T-test; ${\;}^{**}$ Based on the generalized linear model.						

**Table 5:** IOP and glaucoma medication in the first- and second-operated eyes in patients with SCD and without SCD after excluding patients who underwent phacotrabeculectomy.

	**First-operated eye**				**Second-operated eye**			
	**Total**	**Patients without SCD**	**Patients with SCD**	P- Value*	**Total**	**Patients without SCD**	**Patients with SCD**	P -Value*
Preoperative								
IOP	23.50 ± 6.37	22.81 ± 6.87	22.36 ± 3.62	0.71	21.87 ± 5.76	22.0 ± 6.60	21.58 ± 3.53	0.84
Number of medications	3.16 ± 0.68	3.17 ± 0.67	3.27 ± 0.76	0.56	3.24 ± 0.72	3.2 ± 0.71	3.33 ± 0.78	0.61
1 M								
IOP	11.58 ± 0.68	11.20 ± 3.78	13.13 ± 2.95	0.03	11.95 ± 3.38	11.58 ± 3.52	12.75 ± 3.04	0.33
Number of medications	0.13 ± 0.53	0.18 ± 0.61	00.00 ± 00.00	0.03	0.13 ± 0.53	0.19 ± 0.63	0.00 ± 0.00	0.13
3 M								
IOP	11.53 ± 3.63	11.00 ± 3.62	13.59 ± 2.57	0.003	11.97 ± 3.47	11.11 ± 3.55	13.83 ± 2.52	0.02
Number of medications	0.39 ± 0.79	0.31 ± 0.75	0.64 ± 0.79	0.09	0.42 ± 0.76	0.57 ± 0.67	0.75 ± 0.87	0.07
6 M								
IOP	11.81 ± 3.77	11.65 ± 3.75	12.95 ± 2.32	0.13	12.24 ± 3.10	11.69 ± 3.29	13.42 ± 2.35	0.11
Number of medications	0.50 ± 0.86	0.43 ± 0.86	0.73 ± 0.77	0.16	0.53 ± 0.83	0.38 ± 0.80	0.83 ± 0.83	0.12
9 M								
IOP	12.39 ± 3.75	11.98 ± 3.81	13.64 ± 1.84	0.01	12.53 ± 3.13	11.86 ± 3.41	14.00 ± 1.71	0.05
Number of medications	0.79 ± 1.07	0.67 ± 1.12	1.00 ± 0.97	0.22	0.74 ± 1.11	0.58 ± 1.14	1.08 ± 0.99	0.19
12 M								
IOP	12.13 ± 4.30	11.87 ± 4.10	14.54 ± 3.14	0.008	13.16 ± 3.71	12.00 ± 3.36	15.67 ± 3.26	0.003
Number of medications	1.29 ± 1.33	0.96 ± 1.23	1.86 ± 1.17	0.004	1.16 ± 1.22	0.73 ± 1.04	2.08 ± 1.08	0.001
18 M								
IOP	12.54 ± 5.23	12.43 ± 4.78	14.37 ± 5.74	0.18	13.28 ± 4.86	12.00 ± 3.18	16.55 ± 6.86	0.08
Number of medications	1.28 ± 1.28	1.10 ± 1.16	1.87 ± 1.36	0.03	1.31 ± 1.23	0.96 ± 1.06	2.22 ± 1.20	0.007
24 M								
IOP	14.67 ± 6.14	14.63 ± 5.70	15.19 ± 5.60	0.73	14.87 ± 5.17	14.13 ± 4.53	16.78 ± 6.44	0.20
Number of medications	1.61 ± 1.22	1.35 ± 1.20	2.37 ± 1.09	0.003	1.59 ± 1.29	1.22 ± 1.21	2.56 ± 1.03	0.006
36 M								
IOP	13.33 ± 4.72	13.58 ± 4.60	13.14 ± 3.74	0.75	13.63 ± 4.05	13.60 ± 4.07	13.71 ± 4.30	0.95
Number of medications	2.07 ± 1.23	1.81 ± 1.24	2.50 ± 1.02	0.07	1.89 ± 1.22	1.60 ± 1.23	2.71 ± 0.76	0.01
48 M								
IOP	13.56 ± 2.82	13.50 ± 3.42	15.00 ± 2.94	0.17	14.25 ± 3.79	13.76 ± 3.83	15.43 ± 3.69	0.34
Number of medications	2.26 ± 1.23	2.06 ± 1.15	2.69 ± 0.48	0.01	2.21 ± 1.06	1.94 ± 1.14	2.85 ± 0.38	0.008
60 M								
IOP	13.54 ± 3.17	13.03 ± 3.20	13.83 ± 1.75	0.42	12.95 ± 2.48	12.54 ± 2.70	13.83 ± 1.83	0.30
Number of medications	2.14 ± 1.21	1.96 ± 1.29	2.66 ± 0.49	0.01	2.21 ± 1.13	1.92 ± 1.25	2.83 ± 0.41	0.03
Data are shown in mean ± standard deviation; SCD, serous choroidal detachment; IOP, intraocular pressure; M, month ${\;}^{*}$ Based on the *T*-test.								

##  RESULTS

This study was conducted on 90 eyes of 45 patients who underwent BT. The average age of the patients was 59.8 ± 11.1 years. Among them, 30 (66.7%) were male, and the average follow-up time extended to 4.99 ± 2.95 years. Notably, the right eye was the first-operated eye in 22 (48.9%) patients. Trabeculectomy was the selected surgical procedure in 76 eyes (84.4%), while combined phacoemulsification and trabeculectomy (phacotrabeculectomy) was performed in 14 eyes (15.6%). The distribution of glaucoma types included primary open-angle glaucoma (POAG) in 41 (45.6%) eyes, chronic angle-closure glaucoma (CACG) in 23 (25.6%) eyes, and pseudoexfoliation glaucoma (PEG) in 18 (20%) eyes [Table 1]. The mean duration between the first and second surgeries was 2.35 ± 1.15 years.

The five-year cumulative probability of survival stood at 64.1%, with a mean survival duration of 4.3 ± 0.14 years. Furthermore, the five-year cumulative probability of survival in the first- and second-operated eyes amounted to 61.0% and 67.6%, respectively, accompanied by mean survival durations of 4.36 ± 0.20 and 4.31 ± 0.21 years, respectively (log rank = 0.085, *P *= 0.77) [Figures 1A & 1B].

Our analysis incorporated diverse factors, including gender, glaucoma type, systemic diseases, age, and a history of SCD, all of which could potentially impact the model's outcome. Despite the inclusion of several factors, our results revealed that only SCD emerged as a statistically significant predictor to the survival of trabeculectomy in the second-operated eye (hazard ratio: 5.3, *P *= 0.006), as determined by the Cox proportional hazards model [Table 2].

### IOP and Number of Glaucoma Medications

The mean preoperative IOP was 22.07 ± 6.3 mmHg. Specifically, the mean preoperative IOP in the first-operated eye was 22.31 ± 6.6 mmHg, while in the second-operated eye, it measured 21.84 ± 6.0 mmHg (*P *= 0.73). The average number of preoperative glaucoma medications was 3.20 ± 0.7, with 3.16 ± 0.7 and 3.25 ± 0.7 in the first- and second-operated eyes, respectively (*P *= 0.52) [Table 3].

### Complications

Postoperative complications observed in the first-operated eyes included leakage in two eyes, a shallow anterior chamber in three eyes, hyphema in two eyes, and hypotony maculopathy in one eye. Furthermore, complications in the second-operated eyes comprised leakage in one eye, shallow anterior chamber in one eye, and hyphema in one eye. SCD occurred in 13 patients (28.9%), with 12 patients experiencing SCD in the first-operated eye. Notably, SCD occurred bilaterally in 10 patients, representing a significant proportion (76.9%) (*P* < 0.001).

The average age of patients with SCD was 59.6 ± 12.4 years, compared to 59.9 ± 10.9 years for those without SCD. The two groups, with and without SCD, were comparable in terms of age, gender, and type of surgery (*P *= 0.932, *P = *0.165, *P = *0.654).

All patients experiencing SCD were initially phakic. Trabeculectomy alone was the selected procedure for 22 eyes, while one eye underwent phacotrabeculectomy. SCD occurred uniformly in all patients as an early postoperative complication, manifesting within seven days. Following the occurrence of SCD in the first-operated eye, preventive measures were implemented, including strict control of maximum IOP and systemic hypertension in all patients. Medical management, involving the reduction of topical steroids and the initiation of systemic steroids, proved effective in resolving SCD in 20 eyes. However, three eyes required suprachoroidal drainage as part of their treatment.

### Subgroup Analysis 

In the first-operated eyes, the five-year cumulative probability of survival was 71.7% for patients without SCD and 35.0% for patients with SCD. The mean survival duration was 4.41 ± 0.23 years in patients without SCD and 4.20 ± 0.43 years in patients with SCD (log rank = 2.59, *P *= 0.107) [Figure 1C].

The five-year cumulative probability of survival in the second-operated eyes was 82.8% for patients without SCD and 38.5% for patients with SCD. The mean survival duration was 4.72 ± 0.18 years in patients without SCD and 3.38 ± 0.50 years in patients with choroidal effusion (log rank = 9.68, *P *= 0.002) [Figure 1D].

SCD was identified through fundoscopy and was further confirmed using B-scan ultrasonography. The primary management approach involved medical interventions, incorporating the use of cycloplegic drops, tapering of topical steroids, and initiation of systemic steroids. Surgical drainage was considered for refractory cases and instances of appositional kissing SCD.

In the first-operated eyes, the mean IOP in the first and third postoperative months was significantly higher in patients with SCD compared to those without SCD (*P *= 0.02 and *P* = 0.03, respectively) [Figure 2A]. Similarly, in second-operated eyes, the mean IOP was significantly higher in patients with SCD compared to those without this condition at postoperative months 3, 6, 9, and 12 (*P *= 0.03, *P* = 0.07, *P* = 0.02, and *P* = 0.001, respectively) [Figure 2B]. Furthermore, number of glaucoma medications exhibited significant differences in all intervals throughout the study period, except in the first month [Table 4].

Upon re-analysis after excluding individuals who underwent phacotrabeculectomy, notable changes were observed in the results. In the first-operated eye, a significant difference in IOP occurred between the two groups at months 1, 3, 9, and 12 (*P *= 0.03, *P* = 0.003, *P* = 0.01, and *P* = 0.008, respectively). Similarly, this significant difference was also observed in the second-operated eye at months 3, 9, and 12 (*P *= 0.02, *P* = 0.05, and *P* = 0.003, respectively) [Table 5].

##  DISCUSSION

The primary objective of our study was to investigate the incidence of SCD in the contralateral eye following BT. The observed development of SCD in the first eye may indicate a predisposition to its manifestation in the second eye, suggesting a potential association between these events. Notably, the surgical success of the second-operated eyes was found to be comparable to the outcome of the first eye in cases undergoing BT. The most significant factor influencing the success rate of the second-operated eye was the presence of SCD. Baseline IOP and medication usage also exerted a statistically significant impact on the five-year cumulative probability of survival.

In our study, SCD occurred in 13 out of 45 patients (28.9%), with the complication involving both eyes in 10 cases (*P* < 0.001). This substantial incidence of SCD and its possible bilateral occurrence emphasize the need for careful monitoring and management of this complication in patients undergoing BT.

The findings of our current study are consistent with our previous research which demonstrated that SCD has a lasting impact on the surgical success rate.^[14]^ The present results further confirm and extend our prior findings, highlighting the bilateral occurrence of SCD despite the implementation of standard preventive measures.

The precise mechanism underlying SCD has not been fully elucidated to date. It is postulated that the choriocapillaris plays a crucial role in this context, insofar as its fenestrations allow the movement of proteins and fluid into the suprachoroidal space. However, having a normal IOP serves as a counterforce by preventing the accumulation of fluid in the suprachoroidal area. When this delicate balance is disrupted and IOP falls below the episcleral venous pressure, secondary protein leakage from the choriocapillaris ensues, ultimately leading to the development of SCD.^[15]^

Certain conditions predispose patients to SCD, including choroidal hemangioma, nanophthalmos, Sturge-Weber syndrome, idiopathic elevated episcleral venous pressure, carotid-cavernous fistulas, and dural sinus shunts.^[16]^ Numerous studies have investigated risk factors associated with the occurrence of SCD. In contrast to the findings of the present study, some studies have identified lower postoperative IOP and older age as risk factors.^[17,18,19]^ Berke et al^[20]^ reported that SCD is associated with hypertension, age, and hyperopia. However, in line with our results, Altan et al^[21]^ found no significant correlation between age and SCD.

Iwasaki et al conducted a study involving 97 eyes of 97 patients who underwent trabeculectomy and were followed up for four weeks. According to the results, 16 eyes developed SCD, the type of glaucoma in 12 out of the 16 patients was PEG, and individuals with choroidal detachment exhibited thicker corneas. Furthermore, the difference in pre- and postoperative IOP was more pronounced in patients with choroidal detachment compared to other patients. Consequently, thicker cornea, presence of PEG, and more significant changes in IOP pre- and postoperative were identified as risk factors for choroidal detachment.^[22]^

Various studies have explored the outcomes of BT. In a retrospective study, Mietz et al found no statistical difference in terms of IOP, glaucoma medication, and success rate between the two eyes. However, they observed a higher need for additional procedures, such as needling or excision of Tenon's capsule cysts, in second-operated eyes.^[23]^ Jung et al investigated outcomes in patients who underwent BT and reported a similar success rate between the two eyes.^[12]^ Nevertheless, early postoperative IOP and bleb vascularity were higher in the second-operated eyes. In their study, SCD occurred in the first-operated eye of three patients and in three eyes of the contralateral operated eye, although it remained unclear whether SCD occurred bilaterally in the same patients or not.

Iwasaki et al conducted an evaluation of outcomes between the first- and second-operated eyes in 84 patients undergoing BT. Of these, 81% had POAG, and 19% had PEG. While the success rate between the two eyes was similar, the authors observed worsening outcomes in the second-operated eyes of patients when the interval between surgeries was two months or more. Choroidal detachment was observed in 12 eyes in the first-operated eye and 8 eyes in the second-operated eye, but the difference was not statistically significant.^[13]^ However, it was not clarified whether SCD occurred in the same patients or not.

Kiessling et al reported successful outcomes in the second eye of patients undergoing BT. They found that when the operation on the first eye failed, the chance of success in the second eye was 37%, which aligns with the findings of the present study.^[24]^ Zalish et al also assessed outcomes in the second-operated eye in patients with BT and found that the results of the first-operated eye could predict outcomes in the second-operated eye.^[25]^ Therefore, the incidence of SCD in the first-operated eye may indicate a potential likelihood of encountering a similar complication in the other eye.

Several factors may contribute to these results. The standard management of SCD involves reducing topical steroids. However, ocular hypotony during this period may induce inflammation. Several factors can increase inflammation in the early postoperative period, especially in the presence of SCD. Reduced topical steroids, concomitant hypotony, and certain surgical procedures such as choroidal tap, anterior chamber reformation, or injection can collectively elevate postoperative inflammation. The presence of inflammation can potentially stimulate Tenon's fibroblasts, and subsequent fibrosis could lead to higher IOP secondary to surgical failure. The reduced use of topical steroids may lead to the formation of fibrosis, as their anti-inflammatory effects, which would otherwise inhibit this process, are not present. This fibrosis can negatively impact the overall success of trabeculectomy.^[26,27,28]^

Effective preoperative and surgical strategies are essential for minimizing complications and improving outcomes in trabeculectomy. Initiating or maximizing glaucoma medication before surgery serves as an effective strategy to reduce preoperative IOP. Also, ensuring adequate control of blood pressure and heart rate prior to surgery is crucial in minimizing the risk of SCD. On the other hand, temporary discontinuation of anticoagulant medications may be considered as a precautionary measure to reduce the risk of bleeding complications. Additionally, employing a meticulous surgical technique, including prophylactic sclerotomies in specific situations—especially in cases with elevated episcleral venous pressure—can help minimize both intraoperative and postoperative hypotony. Micro-invasive glaucoma surgery can be explored as an alternative approach in certain cases. Furthermore, avoiding the Valsalva maneuver during and after surgery and discontinuing glaucoma medications postoperatively are effective strategies in decreasing the likelihood of SCD.^[16,22]^

By excluding individuals who underwent phacotrabeculectomy, a considerable difference in IOP over an extended period was observed between eyes with SCD and those without SCD. This variation appears to be linked to a decrease in success rates and higher IOP in eyes that undergo phacotrabeculectomy. Sacchi et al reported that patients undergoing trabeculectomy demonstrated lower postoperative IOP—suggesting a higher success rate—and showed a significantly reduced need for needling compared to patients in the phacotrabeculectomy group.^[29]^ However, other studies have reported similar results between the two groups and no significant difference in success rates.^[30,31,32]^

This study has several limitations, including a small sample size and its retrospective nature, potentially impacting the generalizability of the results. Additionally, we did not investigate important factors influencing SCD development such as blood pressure, anticoagulant use, orbital anatomy, refractive error, and axial length parameters. Future studies with larger sample sizes should strive to gather comprehensive preoperative data to more thoroughly assess the potential associations of these factors.

In summary, the study indicates a potential link between SCD in the first- and second-operated eyes, suggesting that the occurrence of SCD in the first eye might predispose the second eye to a higher risk of SCD. In addition to implementing preventive measures to reduce the risk of SCD in second-operated eyes, future studies should address the effects of lowering topical steroids in managing choroidal detachment. Future research can explore other topical drugs with anti-inflammatory effects or further investigate the behavior of SCD while maintaining the use of steroids for its anti-inflammations and anti-fibrosis role.

##  Financial Support and Sponsorship

None.

##  Conflicts of Interest

None.
